# Investigating gut alterations in Alzheimer’s disease: In-depth analysis with micro- and nano-3D X-ray phase contrast tomography

**DOI:** 10.1126/sciadv.adr8511

**Published:** 2025-01-31

**Authors:** Francesca Palermo, Nicole Marrocco, Letizia Dacomo, Elena Grisafi, Viviana Moresi, Alessia Sanna, Lorenzo Massimi, Marianna Musella, Laura Maugeri, Inna Bukreeva, Fabio Fiordaliso, Alessandro Corbelli, Olga Junemann, Marina Eckermann, Peter Cloetens, Timm Weitkamp, Giuseppe Gigli, Nicole Kerlero de Rosbo, Claudia Balducci, Alessia Cedola

**Affiliations:** ^1^ Institute of Nanotechnology – CNR, Rome, Italy.; ^2^ Istituto di Ricerche Farmacologiche Mario Negri IRCCS, Milan, Italy.; ^3^ Synchrotron ESRF, Grenoble France.; ^4^ Synchrotron SOLEIL, Saint-Aubin, France.; ^5^ Institute of Nanotechnology – CNR, Lecce, Italy.; ^6^ University of Salento, Lecce, Italy.

## Abstract

Alzheimer’s disease (AD), a debilitating neurodegenerative disorder, remains one of the foremost public health challenges affecting more than 30 million people worldwide with the etiology still largely enigmatic. The intricate gut-brain axis, serving as a vital communication network between the gut and the brain, appears to wield influence in the progression of AD. Our study showcases the remarkable precision of x-ray phase-contrast tomography (XPCT) in conducting an advanced three-dimensional examination of gut cellular composition and structure. The exploitation of micro- and nano-XPCT on various AD mouse models unveiled relevant alterations in villi and crypts, cellular transformations in Paneth and goblet cells, along with the detection of telocytes, neurons, erythrocytes, and mucus secretion by goblet cells within the gut cavity. The observed gut structural variations may elucidate the transition from dysbiosis to neurodegeneration and cognitive decline. Leveraging XPCT could prove pivotal in early detection and prognosis of the disease.

## Teaser

X-ray imaging reveals gut structural changes in Alzheimer’s mice, potentially linking gut health to brain degeneration.

## INTRODUCTION

Alzheimer’s disease (AD), the most common form of dementia, is a progressive neurodegenerative disorder characterized by synaptic dysfunction, cognitive decline, and multiple brain alterations including synaptic loss, chronic neuroinflammation, and neuronal cell death (*1*). As the prevalence of this devastating condition continues to rise and attempted therapies to fail (*2*), unveiling the underlying mechanisms and identifying therapeutic targets are paramount.

In recent years, there has been growing evidence supporting a bidirectional communication between the gut and the brain (*3*–*6*), involving various neuroendocrine, immune, and neuronal interconnected pathways. Dysfunction in this axis has been implicated in several psychiatric and neurological disorders including AD (*7*, *8*).

The gut microbiota, which refers to the vast community of microorganisms residing in the intestinal tract, support human health and seems to have profound influences on brain function, cognition, and behavior (*9*).

Scientists have discovered that changes in the gut microbiota composition (namely, dysbiosis) can contribute to AD development and progression (*3*–*6*). Gut bacterial communities differ in mild cognitively impaired and AD patients if compared to cognitively normal individuals (*10*, *11*), which suggests that microbiome composition and metabolism might influence disease onset and/or progression. Theoretically, dysbiosis is characterized by a loss of microbial diversity, the prevalence of dangerous bacteria producing toxic metabolites promoting inflammation, and, consequently, the breakage of the gut/brain barriers. In patients with AD, a reduction in the abundance of the bacterial phylum Firmicutes producing essential metabolites and of serotonin transporting bacterium Turicibacter was found (*12*), whereas the proinflammatory taxa Bacteroidetes, Gammaproteobacteria, Enterobacteriales, and Enterobacteriaceae were augmented (*6*, *13*–*15*). Of note, animal models mimicking AD display similar human gut microbiota changes (*16*–*20*). It is assumed that bad bacteria could contribute to the disease by entering the circulation, reaching the brain, and initiating the classical AD-related cascade of neuropathological events, which includes production of various neurotransmitters and neuroactive substances that negatively influence brain function and behavior together with an up-regulation of inflammatory markers in the central nervous system (*21*). These molecules could initiate the vicious cycle of AD also by eventually promoting β-amyloid (Aβ) accumulation (*21*). Experimental investigations have proved that fecal transplantation from AD subjects or AD mice to healthy mice or rats leads to the development of typical disease features such as cognitive impairment, amyloid deposition, and reduced hippocampal neurogenesis (*6*, *22*–*24*).

Understanding the intricate cross-talk between the gut and the brain in AD has opened possibilities for therapeutic interventions ideally at earlier stages of disease, also thanks to the fact that the gut is a more easily accessible organ compared to the brain. To be noticed that risk factors for AD can be distinguished as nonmutable risk factors (i.e., age, sex, and gene mutations) and mutable risk factors (i.e., lifestyle, obesity, and environment) with gut dysbiosis being a mutable risk factor. On the basis of this, researchers are exploring the potential of modulating the gut microbiota through dietary interventions, probiotics, prebiotics, and fecal microbiota transplantation to restore a physiological microbiota, so to improve cognitive functions and alleviate the pathological processes associated with AD (*25*–*27*).

The main hypothesis behind the contribution of gut dysbiosis toward the development of AD or dementia is that upon dysbiosis, multiple gut changes occur also at structural/cellular/functional level: i.e., increased intestinal barrier permeability [namely, “leaky gut” (*28*)], tight junction loss (*29*), reduced mucus release in the gut lumen, and activation of immune cells (*30*). These changes might be responsible for bacteria escape from the gut lumen and initiation of the pathological cascade. At this regard, however, evidence is still poor and further investigation is needed to better describe and unveil these alterations.

The possibility to identify early gut changes through sophisticated imaging, combined with metagenomic data, might, indeed, alert on the ongoing pathological events predisposing to AD at clinical level, but will also allow to establish which biomolecular mechanisms are behind those changes at preclinical level, thus allowing the identification of therapeutic targets.

This paper presents a groundbreaking application of nano– and micro–x-ray phase-contrast tomography (XPCT) (*31*–*35*) to unveil and investigate various structural and morphological gut alterations on the whole organ, without aggressive tissue manipulation such as sectioning and staining. XPCT enables high-resolution three-dimensional (3D) imaging of soft biological tissues, traditionally considered “invisible” to x-rays, with minimal sample preparation and without the use of additional contrast agents. XPCT is particularly suited for preclinical research in neurological diseases, allowing simultaneous visualization of changes in cell abundance and organization, as well as structural alteration in the vascular network and other morphological features (*36*), enabling comparison of physiological and pathological states of key disease targets (*37*).

A single XPCT volume allows the extraction of information that would be contained in thousands of sequential histological sections. Furthermore, a single XPCT image captures all existing structures without the need for specific markers to highlight particular features.

We here report on nano- and micro-XPCT analysis conducted on ileum fragments derived from two mouse model of familial AD (APP/PS1 and APP23) (*38*) and a mouse model of accelerated senescence, widely used as a sporadic AD mouse model (SAMP8) (*39*). The ileum is part of the small intestine, which is affected in AD mice (*40*, *41*).

High-resolution 3D analysis has provided unprecedented gut details, also allowing to execute qualitative and quantitative reliable analysis of whole tissue slabs, providing evidence of alterations occurring in different mouse models of dementia.

## RESULTS

### XPCT allows analysis of crypt and villus morphology in dementia mouse models

To deeply investigate the morphological changes in the gut of AD mice, through XPCT, we analyzed the ileum villi and crypts of various mouse models: healthy wild-type (WT) mice aged matched (18 months) to APP/PS1dE9 and APP23 AD mice, senescence-accelerated mouse resistant-1 (SAMR1) mice, and littermate controls of senescence-accelerated mouse-8 (SAMP8) frail mice of 11 months of age (*38*, *39*).

**Fig. 1.**
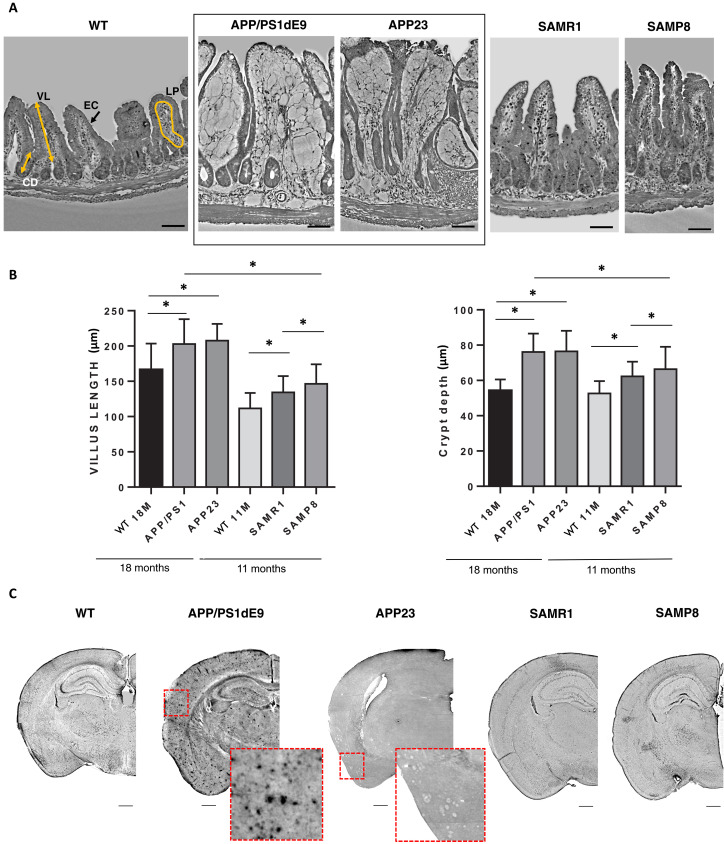
XPCT reveals intestinal villi and crypt changes in AD mutated mice. (**A**) XPCT images of the ileum morphology in different dementia models and their control groups. White pixels represent low-density tissue, while dark pixels represent high-density tissue. Scale bars, 50 μm. The XPCT image of WT helps to define the extension of villus length (VL) and crypt depth (CD) and to illustrate the location/extension of epithelial cells (ECs) and lamina propria (LP). (**B**) Quantification of villus length (left side) and crypt depth (right side) in the different mouse models. Results are shown as mean ± SD. One-way ANOVA *P* < 0.0001; post hoc by Tukey’s post hoc test: *P < 0.0001. (**C**) XPCT of mouse model hemibrains. XPCT images of hemibrains are obtained as *z* projection of minimum intensity over 5 μm. Scale bars, 50 μm. Experiments carried out at the ANATOMIX beamline (SOLEIL) and at the ID19 beamline (ESRF).

The corresponding XPCT images of the ileal villi (Fig. 1A) clearly present impressive differences in shape and density in APP/PS1dE9 or APP23 carrying AD-related human mutations, when compared to the other mouse strains including control groups WT and SAMR1 and SAMP8 mice. The villi of SAMP8, the sporadic AD model, show, instead, a gut conformation comparable to their relative SAMR1 controls. With regard to the villus length, we observe that aged WT mice have longer villi when compared to younger control but, notably, in AD mice villus lengths are even longer than those measured in their age-matched WT littermates, likely indicating that aging might be associated with an increase in villus length, and that the presence of an AD-related pathology exacerbates this change. Small significant differences are detected in SAMP8 mice if compared with SAMR1. Both genotypes exhibit longer villus compared to age-matched WT mice, likely indicating a typical villus conformation of SAM mice which is slightly modified by a frail condition, at least at the age investigated (Fig. 1B). The thickness of the epithelium barrier in APP/PS1 and APP23 is also considerably reduced, while the villus region of lamina propria appears impressively larger.

Morphometric quantification of the crypt also reveals significant differences among mice (Fig. 1B). Both APP/PS1dE9 and APP3 mice show significantly deeper crypts if compared to aged-matched controls. Again, no differences are found at this level between SAMP8 and SAMR1 mice, and also between younger and older WT mice.

Notably, the lamina propria in APP/PS1dE9 and APP23 exhibits lower density, brighter shades of the grayscale, compared to the control groups and SAMP8 mice, while the epithelium displays a more corrugated shape. Our concomitant investigation on corresponding brains of mice whose gut were studied reveals the presence of Aβ deposited plaques only in APP/PS1dE9 and APP23, as expected (Fig. 1C), in the cortex, hippocampus, and thalamus. The two different models are characterized by a different morphology of the plaques. No plaques were detectable in SAMP8 and in WT, SAMR1 mouse brains. While our SAMP8 mice do develop cognitive and motor impairment, as well as brain atrophy and neuropathology (*42*), we found no plaque deposition despite testing multiple specific antibodies for their labeling.

The gut changes described so far, specifically observed only in AD mutated mice, suggest that they may be related to either the APP gene overexpression or the ongoing AD pathology, an issue that deserves further investigation in future studies and throughout longitudinal approaches.

**Fig. 2.**
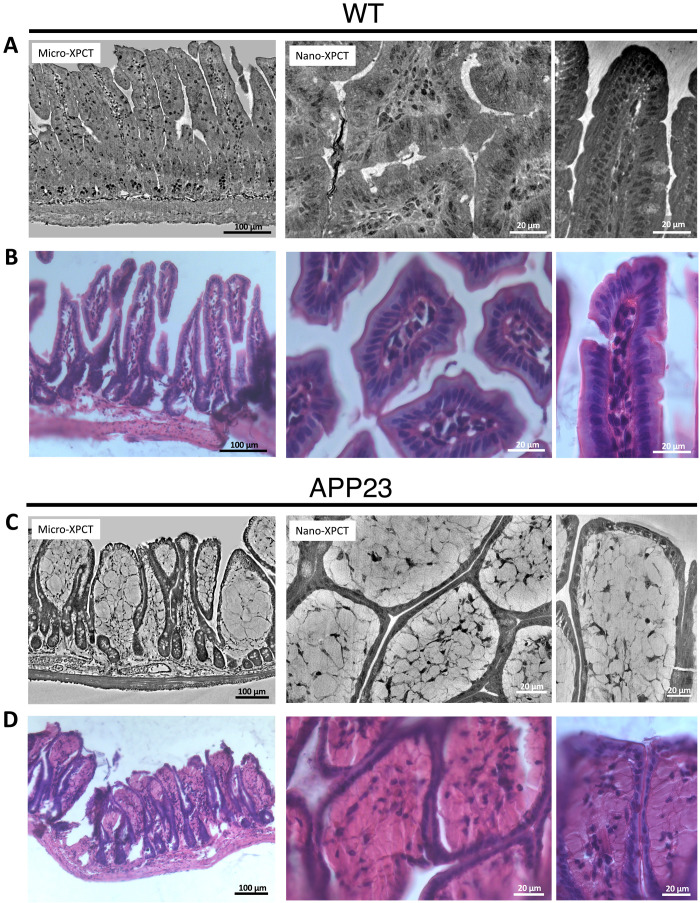
Comparison of micro-XPCT and nano-XPCT with histological sections. (**A**) and (**C**) report micro-XPCT and nano-XPCT slices from a WT mouse as control and a Tg APP23 mouse, respectively. For the nano-XPCT, two virtual sections across perpendicular directions are shown. Similarly, (**B**) and (**D**) report histological sections stained with hematoxylin and eosin at similar scale and across the same directions for comparison with the x-ray findings. XPCT experiment carried out at the ANATOMIX beamline (SOLEIL); nano-XPCT experiment performed at ID16A (ESRF).

The histological analysis performed by hematoxylin and eosin staining validates the features observed in XPCT images (Fig. 2). Ileum of control healthy WT mice shows well-organized villi, characterized by the surface of columnar epithelial enterocytes with a striated border, needed for optimal nutrient absorption, and the lamina propria, a highly cellular loose connective tissue that makes up the core of the villus, containing mostly lymphocytes (Fig. 2, A and B). The ileum of the APP23 mice (Fig. 2, C and D), instead, shows a flat luminal surface of the villi, with a cuboidal striated epithelium and a lamina propria completely disorganized and mostly devoid of cells compared to age-matched control littermates. Round-shape subcellular structures are also observable. Similar features were found for APP/PS1 (fig. S1). Of note, these histological features of AD mouse ileum in part resemble those previously described for the colon of Tg2576 mice, another widely used mouse model of AD (*43*).

### Nano-XPCT allows a multilevel analysis of the mouse gut: Cellular and vascular components

**Fig. 3.**
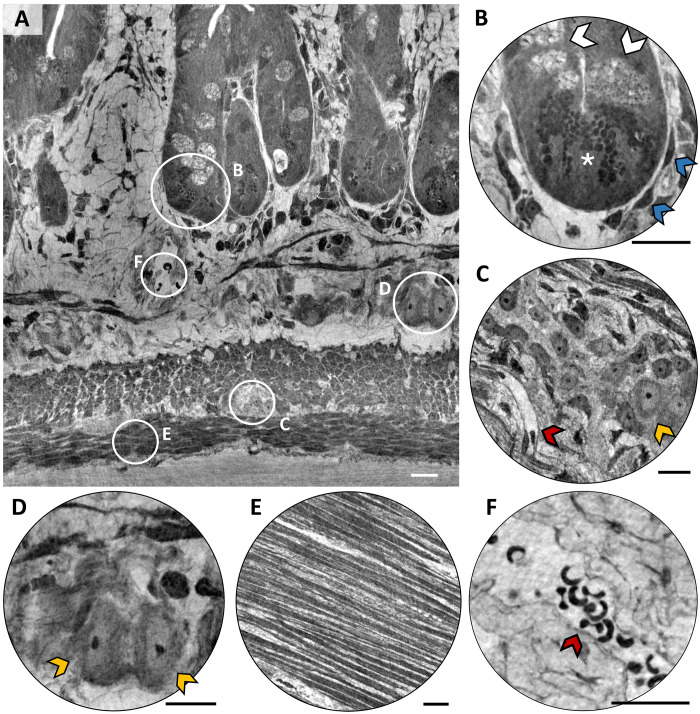
Nanotomography of a mouse ileum. White pixels represent low-density tissue, while dark pixels represent high-density tissue. (**A**) Longitudinal view of the ileum structure at the level of the crypta and the tunica muscularis. The circles indicate the approximate position of the morphological details shown in the figure. (**B**) Bottom of a crypt, where goblet cells are indicated by white arrows, the presence and location of Paneth cells by an asterisk and telocytes by blue arrows. (**C**) Neurons of the myenteric plexus (Auerbach). (**D**) Neurons belonging to the submucosal plexus (Meissner). (**E**) Longitudinal layer of the tunica muscularis. (**F**) Blood vessel with enterocytes (red arrow) that appear as crescent-shaped black structures. Scale bars, 10 μm. Experiment carried out at the beamline ID16A (ESRF).

To further improve the level of detail reachable in gut analysis by XPCT, we also conducted an experiment of nano-XPCT of mouse ileum, with a resolution at the nanometric scale to enable single-cell scale. The high resolution allows to visualize very fine details in the volume and to distinguish individual cells. A representative nano-XPCT image, showing a longitudinal view of the ileum structure at the level of the crypts and the tunica muscularis, obtained from an APP/PS1 sample is shown in Fig. 3A. The different tissues and cellular types are highlighted in circles and shown magnified in the insets of the same figure. The zoom in Fig. 3B shows the bottom of a crypt, where goblet cells are indicated by white arrows, the presence and location of Paneth cells by an asterisk and telocytes by blue arrows. The identification of cells and structures was based on morphological and anatomical considerations, comparison with tabulated histological images (*44*) and with histological sections obtained from the same samples imaged with XPCT (Fig. 2 and fig. S2), and reference to transmission electron microscopy images (*45*, *46*), showing the same biological structures present in our tomograms. Features such as shape, size, and spatial location were used to classify the anatomical details in the XPCT images.

Enteric neurons of the myenteric plexus (or Auerbach’s plexus) are shown in Fig. 3C (transversal view) indicated by yellow arrow. The nanoscale resolution makes it possible to distinguish the internal structure of neurons: The nucleolus appears as a black spot in the center of each body, while the nuclear and cytoplasmic regions can be discriminated, thanks to the different shades of gray corresponding to different material densities. The red arrow indicates a blood vessel containing blood cells (black spots). Figure 3D shows neurons (yellow arrows) belonging to the submucosal plexus (or Meissner’s plexus), which provides secretomotor innervation to the mucosa closest to the lumen of the gut. As for the neurons of Auerbach’s plexus, we can distinguish the internal structure of the cells. The myenteric plexus exists between the longitudinal and circular smooth muscle layers (the latter shown in Fig. 3E) of muscularis externa in the gastrointestinal tract and provides motor innervation to both muscle layers, having parasympathetic and sympathetic input. Meissner’s plexus and Auerbach’s plexus build up the enteric nervous system (*47*). Figure 3F shows a blood vessel, the inside of which appears lighter because it is less dense than the surrounding tissue, filled with erythrocytes, which are individually visible as black crescent-shaped structures.

### 3D reconstruction of Paneth and goblet cells upon XPCT analysis

Paneth and goblet cells belong to the secretory epithelial cell (EC) compartment of the innate immune system. Specifically, Paneth cells, which are highly specialized secretory ECs located at the bottom of the crypt in the small intestine, are nowadays considered as strictly related to the intestinal homeostasis. They release different molecules such as antimicrobials peptide and immunomodulating proteins, which control and modulate gut microbial communities and secrete factors forming a protective biochemical barrier. They increase in number in case of the establishment of an inflammatory condition (*48*).

Goblet cells are one of the cell types in the secretory lineage originating from stem cells, equipped with an efficient biological machinery for mucus secretion, and inhabiting the entire length of the intestine. These cells contribute to intestinal protection through the secretion of different types of mucus serving as a protective barrier. In the small intestine, goblet cells located in the crypts secrete a dense mucus functioning as a diffusion barrier for antimicrobial proteins and peptides keeping bacteria at a distance, whereas goblet cells placed in the villi rapidly secrete a type of mucus that can be easily removed to allow nutritional uptake. When goblet cell function is compromised, unprotected regions of the gut epithelium appear, allowing bacteria to escape from the gut lumen, enter the circulation and initiate inflammatory events (*49*). Protein misfolding–related diseases, such as AD, could result in altered production and apoptosis of goblet cells, in turn affecting mucus properties. However, an increase in the number of both Paneth and goblet cells, as well as higher mucus release, has been also described in AD mice (*50*). This contrasting evidence encourages sophisticated analysis of Paneth and goblet cells in AD mice, as well as mucus release, which could be instrumental to more precisely unveil AD-related gut alterations.

**Fig. 4.**
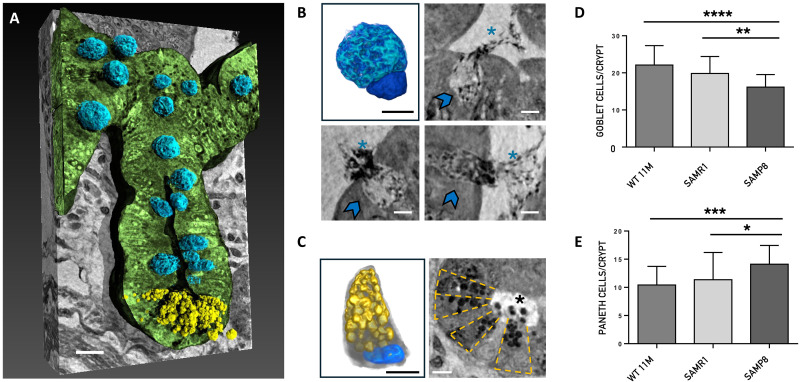
Nano-XPCT analysis of the Paneth and goblet cells. (**A**) Representative 3D rendering of the longitudinal view of one crypt of SAMR1 mouse. The epithelial layer of the crypt has been rendered in green. The Paneth cells are colored in yellow and the goblet cells in blue. Scale bars, 5 μm. (**B**) and (**C**) show 3D renderings and nano-XPCT close-ups of goblet and Paneth cells, respectively. The same color code of (A) has been used for the 3D renderings shown in (B) and (C). In detail, the goblet cell nucleus is colored in dark blue and the apical portion, expanded with mucin-secreting granules, which extends into the intestinal lumen, is rendered in light blue. The Paneth cell presents the typical pyramidal shape with basally situated nucleus (blue) and prominent apical granules (yellow) that occupy most of its cytoplasmatic region. Nano-XPCT close-ups in (B) show goblet cells (indicated by arrows) secreting mucus (asterisks) in the intestinal lumen. Scale bars, 2.5 μm. In (C), transversal view of a crypt in which the Paneth cells are arranged in a radial pattern (highlighted by the dashed-line boxes). The release of the antimicrobial granules (black dots, asterisk) into the lumen is visible. Scale bars, 5 μm. (**D** and **E**) Quantification of goblet and Paneth cells in the crypts. Results are obtained on 30 crypt per mouse (*n* = 1) and are shown as mean ± SD. One-way ANOVA *P* < 0.0001; post hoc by Tukey’s post hoc test: *P < 0.02, **P < 0.01, ***P < 0.001, and ****P < 0.0001.

XPCT analysis revealed both Paneth and goblet cells in great detail, allowing precise quantification of their abundance. Figure 4A shows a representative 3D rendering of the longitudinal view of one crypt of SAMR1 mouse. The epithelial layer of the crypt is rendered in green; the dark fissure in the center of the structure corresponds to the crypt lumen; Paneth cells are colored in yellow and goblet cells in blue. The same color code has been used in the 3D renderings in Fig. 4 (B and C), which show a goblet and a Paneth cell, respectively. In detail, the rendering in Fig. 4B shows a single goblet cell displaying its characteristic polarized morphology with the nucleus (dark blue) located at the narrow base of the cell and a large oval apical portion (light blue) (expanded with mucin-secreting granules) extending into the intestinal lumen. The Paneth cell in Fig. 4C presents the typical pyramidal shape, with basally situated nucleus (colored in blue) and prominent apical granules (yellow) that occupy most of its cytoplasmatic region.

It is intriguing to also note how clearly XPCT allowed to visualize, and thus analyze, goblet cell mucus or Paneth cell antimicrobial granule release in the gut lumen (Fig. 4, B and C, asterisks).

Again, to prove the possibility to make quantitative analyses, we show in Fig. 4 (D and E) an example of data obtained by quantifying the cells of 30 crypt per mouse from 3D reconstructed gut slices of solely one mouse per genotype of the SAM groups versus an aged-matched WT mouse. Quantification allowed to unveil relevant changes in both goblet and Paneth cells in SAMP8 mice, which will be further investigated in larger experimental mouse groups.

### XPCT allows identification and analysis of telocytes

**Fig. 5.**
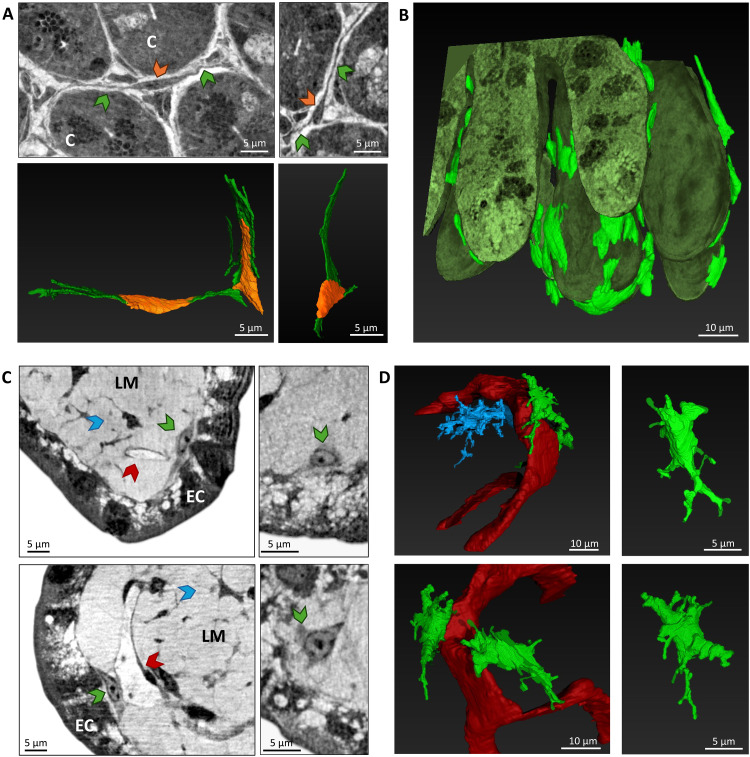
Nano-XPCT analysis of telocytes. (**A**) Nano-XPCT images of telocytes located around ileal crypts (indicated by the capital letter “C”) in transversal view and the corresponding 3D renderings. The figures help to illustrate the structure of telocytes: The cell body is indicated by orange arrows in the XPCT images and it was rendered in the 3D images with the same color; the cell processes (telopodes) are indicated and rendered in green. (**B**) 3D renderings of ileal crypts surrounded by telocytes (bright green) in longitudinal view. (**C**) Nano-XPCT close-ups of the tips of ileal villi in longitudinal and transversal view. Telocytes (green arrows) are located in the lamina propria (LM) close to the EC layer and blood capillaries (red arrows). Dendritic-like cells are indicated by blue arrows. (**D**) 3D renderings of telocytes located in the villus tip. The telocytes are colored in green, the blood capillary in red, and cells compatible with dendritic cells are rendered in blue. Experiment carried out at ID16A (ESRF).

Telocytes (*51*, *52*), a distinctive type of interstitial cells, are unique mesenchymal cells featured by numerous long cytoplasmic extensions, called telopodes, extending hundreds of micrometers away from the cell body. To prove the enormous potential of XPCT in providing structural, morphological, and cellular details, in Fig. 5A, we show two XPCT images of telocytes (upper panel) and two images of segmented and 3D reconstructed telocytes from different view sides (lower panels); in Fig. 5B a 3D reconstruction of telocytes clearly show their placement adjacent to the crypts. Telocytes are normally quiescent state cells and do not divide until environment perturbations occur, which they react to rapidly expanding in the attempt to restore a physiological condition (*51*). Although these cells remain largely unknown in terms of role and mechanisms of actions, there is growing interest in better understanding their involvement in health and pathology (*52*). At the state of the art, it is known that different telocyte subtypes exist adapting to the different sites in which they reside by performing different functions (*52*). They are mainly involved in the differentiation and proliferation of intestinal crypt stem cells and interact with other cell types (macrophages, smooth muscle cells, and other immune cells), either by establishing cell membrane contacts (*52*) or by producing exosomes, for various purposes, e.g., mediating chemotaxis of immune cells and organ repair. It has been reported that telocytes themselves can act as immune cells (*53*).

To date, telocytes are distinguishable only through transmission electron microscopy, whereas it is still debatable which markers are reliable for their identification through immunohistochemistry, and at the state of the art, at least three different antibodies must be used concomitantly (*51*). In contrast, XPCT successfully allowed to well distinguish telocytes on the basis of their distinctive cell morphology and their placement. As observable in the XPCT Fig. 5A, telocytes (indicated by orange arrows) present very long and very thin prolongations, namely, the telopodes (indicated by green arrows), which can be clearly seen with XPCT but are mostly below the resolving power of light microscopy. Figure 5A shows telocytes surrounding the epithelium of the crypts (upper panels) and the 3D renderings of isolated telocytes (lower panels). Using the same color code as in the XPCT tomograms in the upper panels, two different colors are used in the 3D renderings to distinguish the soma and telopodes of the telocytes: The soma are rendered in orange and the telopodes in green. As displayed in the 3D rendering of Fig. 5B, telocytes (colored in bright green) are aligned along the crypt-villus axis, in close association with the intestinal epithelium and blood vessels, forming a continuous network along the basal side of the entire crypt-villus epithelium (dark green) (*51*). Figure 5C displays XPCT close-ups of the tips of the ileal villi, where the telocytes (indicated by green arrows) are in close contact with the intestinal epithelium (EC) and blood vessels (red arrows). 3D renderings of telocytes in the villus tip are shown in Fig. 5D, allowing a better visualization of the position of these cells (in green) in relation to blood capillaries (colored in red) and to other cells likely to be compatible with dendritic cells (in blue).

### Lymphatic follicles

**Fig. 6.**
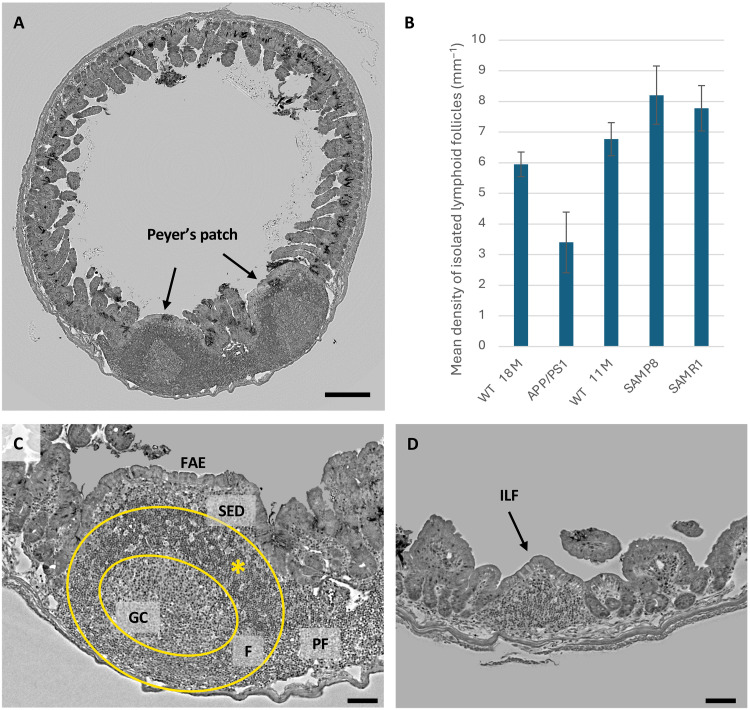
XPCT analysis of lymphoid follicles. (**A**) XPCT image of a section of ileum, where two follicles of a Peyer’s patch (PP) are visible (black arrows). Scale bar, 200 μm. (**B**) Mean density for ileum length unit of lymphoid follicles. Results are shown as mean ± SD. (**C**) XPCT image of a PP. The spatial and contrast resolutions provided by XPCT allow to distinguish the regions which compose the PP. The figure helps to illustrate the location/extension of germinal center (GC), follicular area (F), subepithelial dome (SED), follicle-associated epithelium (FAE), and parafollicular area (PF). Note the densely packed lymphoid cells (asterisks) within the follicular area. (**D**) Isolated lymphatic follicle (ILF). (C) and (D) are on the same scale to allow the comparison of size. Scale bars, 50 μm. XPCT images were obtained as *z* projection of minimum intensity over 3 μm. The experiment was carried out at the ANATOMIX beamline (SOLEIL).

The ability of XPCT to allow 3D reconstruction of the entire organ allowed us to study the presence and the conformation of Peyer’s patches (PPs) and isolated lymphoid follicles (ILFs), throughout the ileum. PPs are recognized as a group of well-organized lymphoid follicles positioned inside the lamina propria and submucosa of the distal portion of the small intestine (ileum, jejunum, and sometimes in the duodenum). PPs and ILFs have been known as the gut defender of the intestinal wall responding to harmful bacteria and parasites; indeed, they are recognized as the immune sensors of the intestine due to their ability to translocate luminal antigens and bacteria across the follicle-associated epithelium (FAE) to the underlying immune cells, contained in the germinal center (GC) and in the subepithelial dome (SD). The immune cells can inhibit or activate the immune response, resulting in either tolerance or systemic immune cell response (*54*) within the mucosa. PPs are characterized by follicle centers and distinct cellular regions overlaid by a specialized epithelium that lacks crypts or villi. In 5XFAD AD mice, elevated levels of inflammatory cytokines and chemokines including interleukin-12 and macrophage inflammatory protein-1α in PPs have been reported (*55*). XPCT also allowed to well identify PPs and clearly distinguish their subcompartments. Figure 6A shows an XPCT image of the section of ileum, where two follicles of a PP are visible, pointed by the black arrows. To illustrate the difference in size between these structures, Fig. 6 (C and D) shows XPCT images of a PP and a ILF in the ileal villi. An increased numbers of PPs and ILFs well correlate with the development of prions disease, with the M cells up-taking the invaded prions, and the PPs acting as replicating centers of the prions (*56*). ILF analysis in AD might thus be highly informative of gut environment alteration and of an ongoing inflammatory condition.

Also, at this level, XPCT allowed to identify PPs and well distinguish the inner structural features indicated by the arrows in Fig. 6C: GC, SD, FAE, and parafollicular area (PF).

Quantification of the number of ILFs has been expressed as the mean density of ILFs for ileum length unit. The ILF density of each group analyzed is shown in Fig. 6B. Only ileal segments fully visible in the tomographic volume were considered to avoid underestimation of ILF density. We did not include intestinal tract that were partially outside the tomographic field of views or damaged due to preparation.

## DISCUSSION

AD has been mainly considered for decades as a “brain pathology,” with the overproduction of Aβ and aggregated species formation and deposition as the mostly recognized primary cause. The multitude of Aβ-centric clinical trials failing throughout years has, however, progressively indicated that diagnosis comes at stages when the central nervous system is so severely compromised at multiple levels that single target therapy most likely will not succeed (*57*, *58*). This increasing awareness has encouraged the identification of earlier brain and plasma biomarkers with the main idea to recognize initial disease stages so to timely intervene. Recent evidence, however, has shed light on the involvement of peripheral organs in AD development and neuropathology progression, such as the eyes and the gut (*59*, *60*).

The gut-brain axis receives particularly attracting attention, because of evidence highlighting an intimate, mutual influence between the gut and the brain through a bidirectional communication. The main accepted theory is that dysbiosis, meant as a pathological change in gut microbiota composition, would predispose to the development of AD, and that preservation/restoration of a more physiological gut microbiota with prebiotics, probiotics, or gut microbiota transplantation will prevent its development or slow down its progression, respectively (*7*, *61*).

One of the mostly assumed mechanisms linking gut dysbiosis to an increased risk of AD is that pathological bacteria predominating during dysbiosis will produce gut structural alterations, likely driven by inflammatory-related events: augmented lipopolysaccharides, pro-inflammatory cytokines, T helper cells and monocytes, and increased gut barrier permeability. These events will then favor bacteria escape from the gut lumen, which by entering the circulation, together with the overproduced inflammatory cytokines, will cause blood brain barrier breakage and eventually bacteria invasion inside the brain (*62*, *63*). Bacteria invasion would promote the initiation of neuroinflammation leading to abnormal Aβ production. Data from AD brain subjects reported the presence of gut bacteria around Aβ deposited plaques in the brain parenchyma associated with an increase of pro-inflammatory cytokines in plasma and brain (*13*). Gut transplantation from AD subjects or mice into healthy mice reproduces neuropathology signatures of AD such as cognitive impairment, amyloid brain deposition, and neuroinflammation (*6*, *62*). A possible association of the gut dysbiosis with Aβ pathway in AD has been hypothesized, providing a potential alternative approach to alter the brain Aβ load (*64*).

At the state of the art, however, most of the research is focused on dysbiosis, gut microbiota characterization, and bacteria composition. Omics analysis including 16 ribosome DNA (rDNA) amplicon and metagenomic sequencing look for association between microbial composition changes and brain dysfunctional and neuropathological signatures (*65*).

No considerable efforts are, instead, devoted to the investigation of gut structural changes, which might possibly explain, or even predict, microbial gut escape from the lumen and the risk of disease initiation. To address this need, we here interrogated nano- and micro-XPCT on their ability to deeply investigate in a 3D fashion the anatomy and cellular components of the mouse ileum in different mouse models of AD-dementia. Our results provide compelling evidence on the enormous power of XPCT to visualize the intestinal environment in 3D with an incredible level of detail and resolution, allowing even subtle intestinal changes to be detected. XPCT permitted to distinguish specific structural changes of the crypts and the villi in the ileum of APP/PS1dE9 and APP23 transgenic (Tg) mice, which were not detectable in the spontaneous SAMP8 mouse model of accelerated aging, although featured by AD-like cognitive symptoms and brain alterations (*42*). XPCT also allowed to concomitantly visualize and distinguish neurons of the myenteric plexus (Auerbach), from neurons belonging to the submucosal plexus (Meissner), together composing the enteric nervous system, as well as the longitudinal layer of the tunica muscularis. Blood vessels were also clearly visible together with the presence of circulating erythrocytes. It is to be emphasized also that through XPCT, we have been able to easily identify telocytes placed along the ileum villi and crypts. This cell subtype is still underinvestigated, but telocytes seem to represent a therapeutic resource which urgently calls for further investigation. Evidence, indeed, indicates that in a pathological context, telocytes decrease in their number, whereas their implantation in the injured tissue would stimulate the activity of regenerative stem cell niche (*66*). This hypothesis implies that their monitoring might be highly informative of an ongoing pathology, alerting on the need of a prompt intervention. The main difficulty, however, is that their identification through immunohistochemistry is extremely difficult, because of the lack of specific markers, requiring multiple labeling to increase the certainty of their identity. So far, only electron microscopy allowed precise telocyte visualization. We here demonstrated that XPCT, by reproducing the entire gut environment in 3D, represents a breakthrough, allowing to analyze and quantify, in an enormous number of acquired images, the number of telocytes together with the length and number of their telopodes with 100% certainty. Furthermore, this technique permitted to clearly visualize and reconstruct in 3D, gut cells covering crucial gut functions, such as goblet cell involved in mucus secretion and gut protection, Paneth cells guaranteeing gut homeostasis preservation and defense, and Payer’s patches, immune sensors of the intestine eliciting an immune response in case of pathogen or danger presentation. Also, at this cellular level, XPCT allows to collect and analyze thousands of acquired images and provides a large-scale analysis of cell number and morphology in health and disease.

In conclusion, we have proved that XPCT represents a breakthrough for a systematic and comprehensive analysis of the gut, instrumental to address and define which changes characterize AD subjects throughout pathology progression, and in association with cognitive dysfunction and brain neuropathological changes.

## MATERIALS AND METHODS

### Samples

The present study was performed on excised brains and intestines from three mouse models of dementia: (i) APPswe, PSEN1dE9 mice [B6C3-Tg(APPswe,PSEN1dE9)85Dbo/Mmjax mice] expressing a chimeric mouse/human amyloid precursor protein with the Swedish mutation (Mo/HuAPP695swe) and a mutant human presenilin 1 (PS1-dE9): *n* = 3 mice, 18 months old (Jackson Lab, USA); (ii) APP23 containing the Swedish double mutation on APP (APP751*K670N/M671L): *n* = 3 mice, 18 months old; (iii) SAMP8: *n* = 8 mice, 11 months old (Envigo, Italy); and (iv) SAMR1: *n* = 5 mice, 11 months old (Envigo, Italy).

Additional samples from C57BL/6 WT mice were used as age-matched controls: *n* = 7 mice, 11 months old; and *n* = 3 mice, 18 months old. Table 1 summarizes the models and the number of samples analyzed in the present work.

**Table 1.** Analyzed samples.

Model	Age	Description	Number of samples
APP/PS1	18 months	Familial AD	3
APP23	18 months	Familial AD	3
C57BL/6	18 months	Aged NTg	3
SAMP8	11 months	Sporadic AD	8
SAMR1	11 months	Sporadic AD control	5
C57BL/6	11 months	Age-matched control	7

Both APP/PS1 (*67*) and APP23 (*68*) mice are animal models widely used to study genetic AD. They carry typical human AD mutations and develop an AD phenotype including cognitive impairment and Aβ plaque deposition in the brain, neuronal damage, neuroinflammation, and brain atrophy. APP23 mice develop cognitive impairment at 6 months of age, while APP/PS1 mice develop that at 11 to 12 months of age.

SAMP8 is a mouse model of accelerated senescence that was established through phenotypic selection (*69*). SAMP8 mice specifically develop dementia, reflecting typical brain alterations of AD (immune alterations, oxidative stress, mitochondrial dysfunction, neurodegeneration, and brain atrophy) (*70*), together with deficits in learning and memory (*39*). This model, often associated with sporadic AD, is usually compared with SAMR1 mice, which show normal aging process (*71*).

**Fig. 7.**
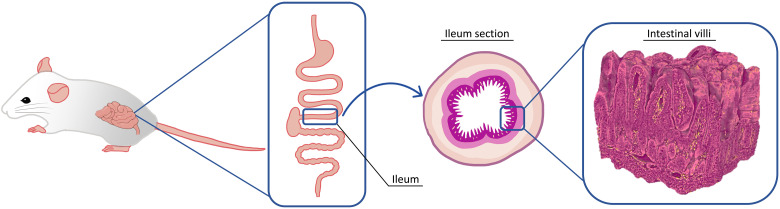
Schematic illustrative picture showing the localization of the ileum in the mouse gastrointestinal tract. The last panel on the right displays the 3D rendering of a portion of intestinal villi obtained from XPCT images.

All animals were housed in a specific pathogen–free facility in groups of four in standard mouse cages containing sawdust with food (2018S Envigo diet) and water ad libitum, under conventional laboratory conditions (room temperature, 20° ± 2°C; humidity, 60%) and a 12/12-hour light/dark cycle. The IRFMN adheres to the principles set out in the following laws, regulation, and policies governing the Care and Use of Laboratory Animals: Italian Governing Law (D.lgs 26/2014; Authorization n.19/2008-A issued 6 March 2008 by Ministry of Health), Mario Negri Institutional Regulations and Policies providing internal authorization for persons conducting animal experiments (Quality Management System Certificate – UNI EN ISO 9001:2015 – Reg. N° 6121), the NIH Guide for the Care and Use of Laboratory Animals (2011 edition), and EU directives and guidelines (EEC Council Directive 2010/63/UE). The statement of Compliance (Assurance) with the Public Health Service Policy on Human Care and Use of Laboratory Animals has been reviewed (9 September 2014; Animal Welfare Assurance #A5023-01). All animals were managed in accordance with European directive 2010/63/UE and with Italian law D.l. 26/2014. The procedures were approved by the local animal health and ethical committee and were authorized by the national authority (Istituto Superiore di Sanità; authorization numbers: 418/2021-PR and 624/2023-PR). All efforts were made to reduce the number of animals by following the 3R’s rule. Mice were euthanized under CO_2_ inhalation. Brains and intestine were excised. Intestine was cut to select the ileum (Fig. 7). Samples were dehydrated through a graded ethanol series (70/95/100%), put in propylene oxide, and subsequently included in paraffin wax. No contrast agent has been used in the preparation of samples prior x-ray imaging.

For the micro-XPCT experiments, the whole paraffin-embedded samples were scanned to obtain the tomographic volumes of the entire organs. The dimensions of the paraffin-embedded brains were about 0.8 × 0.5 × 1 (width-depth-height) cm^3^, whereas the ileum measured about 0.2 × 0.2 × 0.8 cm^3^. For the nano-XPCT experiment at the ID16A beamline, 500-μm biopsies were punched out from identified regions of interest of the samples already measured at the microscale, guided by tomographic prescan acquired at the lower resolution. For tomographic imaging, the paraffin-embedded tissue biopsies were then placed on metal pins.

### Micro-XPCT

The XPCT experiments were performed at (i) the ANATOMIX beamline of Synchrotron SOLEIL (Paris, France) (*72*) and at (ii) the beamline ID19 of the European Synchrotron Radiation Facility (ESRF, Grenoble, France), in free-space propagation mode.

(i) The XPCT experiment at ANATOMIX beamline was performed with a filtered white beam. The measurements were performed at two different resolutions: scans with an effective pixel size of 3.07 μm were taken with an energy spectrum peaked around 30 keV, at a propagation distance between sample and detector of 1 m, and an exposure time of 150 ms per projection radiograph. Higher-resolution data were taken at an effective pixel size of 0.65 μm, central photon energy around 20 keV, a propagation distance of 20 mm, and an exposure time of 100 ms per projection angle. In both cases, the detector was an indirect system consisting of a lutetium aluminum garnet scintillator coupled via a lens system (magnifications 2.1× and 10×, respectively, for the two resolutions) to a Hamamatsu Orca Flash 4.0 camera [sensor type, complementary metal-oxide semiconductor (CMOS); sensor array size, 2048 × 2048 pixels; physical pixel size, 6.5 μm; and 16-bit nominal dynamic range]. The tomography was produced by means of 4000 projections in extended field-of-view (FOV) mode, an acquisition method, which allows to almost double the effective horizontal width of the FOV of the detector. The rotation axis is moved close to either left or right side of the FOV and a dataset of projections, having size equal to the detector FOV, is collected over 360°. After properly stitching the sinograms, the reconstruction procedure can be performed as usual. Data preprocessing, phase retrieval, and tomographic reconstruction were performed using the standard data processing pipeline at the beamline, based on a Python script as a user front end and for preprocessing and the PyHST2 software package (*73*) as the backend for the tomographic reconstruction.

(ii) Data acquisition at ID19 was carried out using pink beam with an energy peaked around 26.5 keV. The sample-detector distance was set at 10 cm. The detector had an effective pixel size of 0.65 μm. The experiment was carried out recording 3600 projections in extended FOV mode covering a total angle range of 360°. The acquisition time for each angular position was 150 ms. Data preprocessing, phase retrieval, and tomographic reconstruction were performed with Tomwer software (*74*) provided by the ESRF.

In both the experiments, phase retrieval was performed by using Paganin’s algorithm (*75*), since we acquired a single image per angle—out of focus, on a plane placed at the Fresnel’s distance, where the phase signal is maximum (*76*)—in free-space propagation mode, and the radiation wavelength and material density met the requirements. This method allows the simultaneous extraction of phase and amplitude of the wave as the ratio of the phase term over the absorption term. Tomographic reconstruction of the 3D volumes was performed from the phase-retrieved angular projections using the Filtered Back Projection algorithm.

### Nano-XPCT

We imaged the samples of ileum at higher resolution exploiting nano-XPCT. The experiment was carried out at the Nano-Imaging ID16A beamline of the ESRF (Grenoble, France) with an effective pixel size of 80 nm. A pair of multilayer-coated Kirkpatrick-Baez optics was used to focus the x-rays (∼30 nm) at 17 keV. The sample was put in the divergent beam downstream of the focus to produce magnified phase-contrast images. The projection geometry also allows zooming into specific regions of a large sample by combining scans with different magnifications and FOV (*77*, *78*). By measuring the Fresnel diffraction patterns at four different effective propagation distances, the phase maps of the sample can be retrieved via holographic reconstruction, this so-called phase-retrieval procedure (*79*) being implemented using GNU Octave software. Magnified radiographs were recorded onto an x-ray detector using a scientific CMOS camera from XIMEA GmbH, with a GSENSE6060 sensor from Gpixel, 6144 × 6144 pixels with each 10 μm edge length. For one tomography scan, 2000 projections were acquired with 0.2 s exposure time. Tomography scans at four different focus-to-sample distances, i.e., four different Fresnel numbers, were acquired to complete one holotomography scan. The tomographic reconstruction was obtained with ESRF PyHST software package (*80*).

### XPCT image analysis

Image analysis and segmentation were performed using ImageJ. 3D rendering images were obtained using the high-end software VGstudio Max. Before extracting qualitative and quantitative information, images were prepreprocessed to eliminate or mitigate artifacts due to experimental conditions or computational reconstruction.

In the XPCT and nano-XPCT images, the shades of gray are proportional to electron density, with black corresponding to the highest value of the density spectrum, whereas white corresponds to the lowest value, hence to features of lowest density.

To enhance the contrast and visualize structures developing in 3D and therefore lying on different tomographic slices, we exploited the *z* projection of minimum intensities, which consists in projecting on the visualization plane the voxels of a set of continuous slices. Each pixel of the output image contains the minimum value found along the axis perpendicular to that pixel. Minimum intensity projection enlightens high-density structures such as cell nuclei and amyloid plaques.

Morphometric villus parameters (crypt depth and villus length), density of goblet and Paneth cells, and density of ILFs were quantified twice independently by three observers (authors F.P. and N.M., F.P. and M.M., and F.P. and N.M., respectively) to improve the reliability of measurements. To limit measurement errors, the length of the villi was measured at the point of maximum elongation, as it was possible to work on the virtual 3D volume. The same consideration is valid for the quantification of crypt depth. About 300 villi per sample were analyzed.

### Histological analyses

Ileum portions from WT and APP23, included in paraffin, were cut by using a Leica microtome (Leica, Wetzlar, Germany) in 10-μm-thick sections. Hematoxylin and eosin (Sigma-Aldrich, Saint Louis, MO, USA) staining was performed according to the standard manufacturer’s instructions. In brief, after deparaffinization to water, slides were stained for 10 min with Mayer’s hematoxylin solution. Slides were then washed under tap water for 10 min and counterstained with eosin Y solution for few seconds. After removing the excess of eosin, sections were dehydrated, and cover slips were mounted with Eukitt mounting medium. Representative pictures were acquired by using an Axioplan microscope (Zeiss, Germany) with Axiocam 503 color and ZEN 3.3 (blue edition) software.

### Statistics

The differences between mouse models were analyzed by considering all objects measured in the same genotype as statistical sample. “*n*” the total number of objects measured per group. After verifying the normal distribution of the data and their homoscedasticity by means of Levene’s test, data were analyzed with one-way analysis of variance (ANOVA), followed by Tukey’s post hoc test or Student’s *t* test as post hoc test. All values were expressed as mean ± SD.
