# Supplementary material for: Investigating gut alterations in Alzheimer’s disease: In-depth analysis with micro- and nano-3D X-ray phase contrast tomography
Source: Sci Adv. 2025 Jan 31;11(5):eadr8511. doi: 10.1126/sciadv.adr8511 (PMC11784835; doi:10.1126/sciadv.adr8511)
Supplement: Supplementary file 1 — Figs. S1 and S2 Legends for tables S1 to S4 Legend for movie S1 [file sciadv.adr8511_sm.pdf]

Science Advances
AAAS

Supplementary Materials for

Investigating gut alterations in Alzheimer's disease: In-depth analysis with micro- and nano-3D X-ray phase contrast tomography

Francesca Palermo *et al.*

Corresponding author: Alessia Cedola, alessia.cedola@cnr.it; Claudia Balducci, claudia.balducci@marionegri.it

Sci. Adv. **11**, eadr8511 (2025)
DOI: 10.1126/sciadv.adr8511

The PDF file includes:

Figs. S1 and S2
Legends for tables S1 to S4
Legend for movie S1

Other Supplementary Material for this manuscript includes the following:

Tables S1 to S4
Movie S1

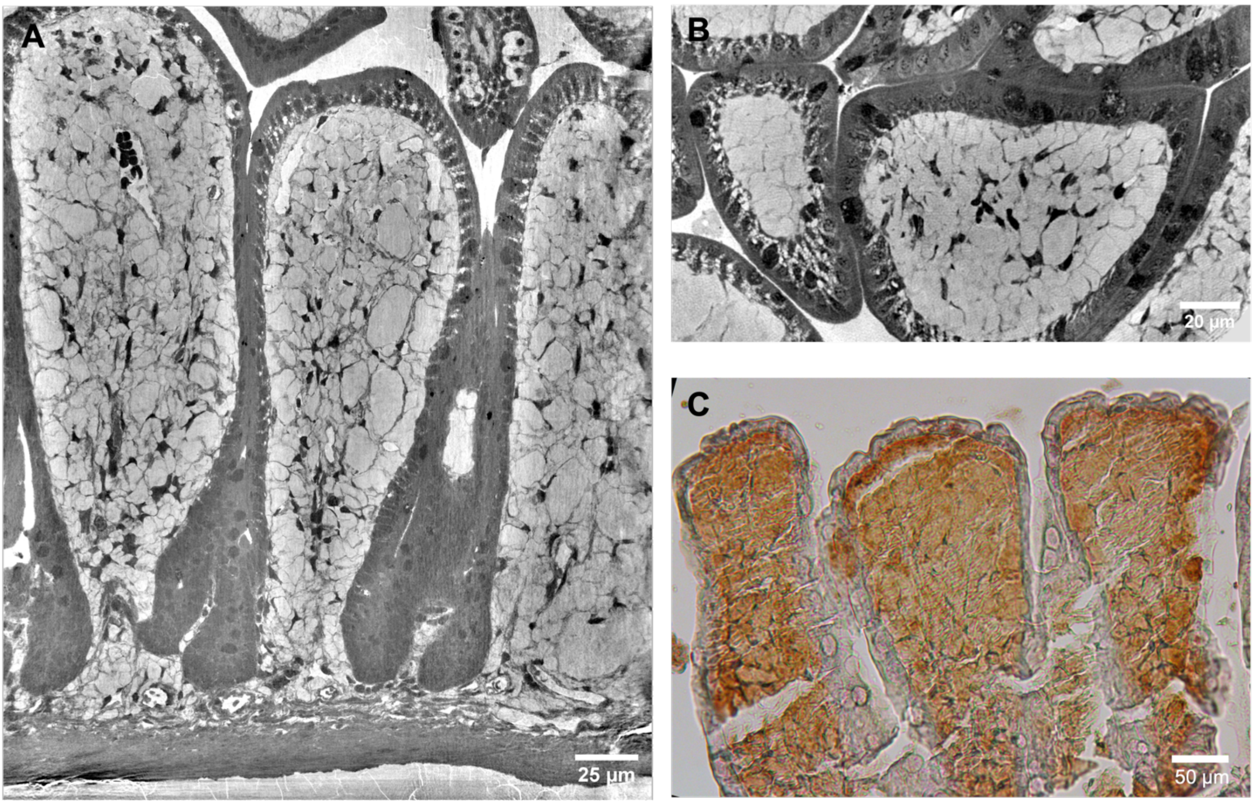

Figure S1. **Nano-X-ray phase contrast tomography (XPCT) and histological section of APP/PS1dE9.** A) and B) report nano-XPCT images from transgenic APP23 mice, in longitudinal and transversal view, respectively. C) The reported histological section was stained with hematoxylin and Beta-III Tubulin Antibody. XPCT experiment performed at ID16A (ESRF). In these XPCT images, the shades of the greyscale are proportional to the electron density of the tissues, with black corresponding to the highest value of the density spectrum, whereas white corresponds to the lowest value, hence, to features of lowest density.

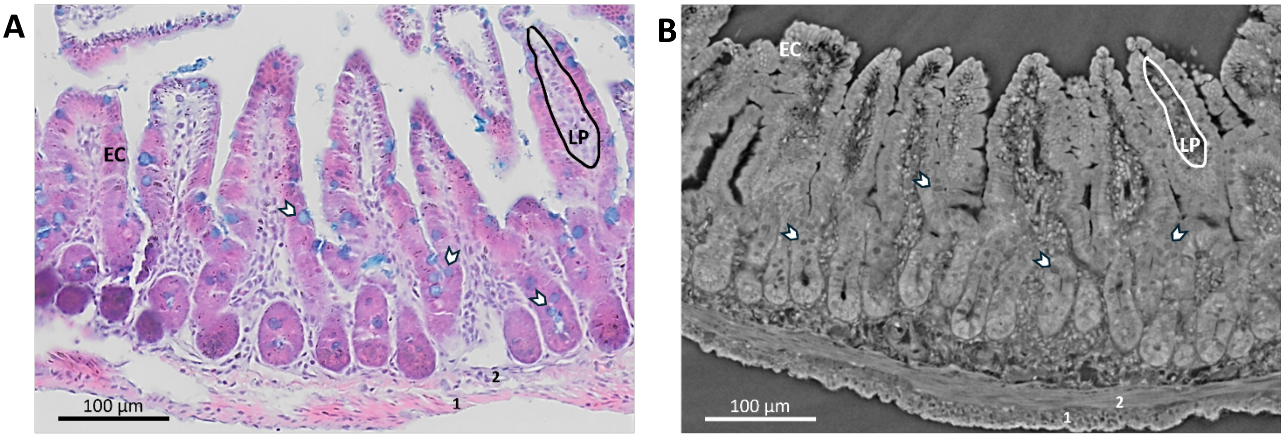

Figure S2. **Comparison of histology and micro-XPCT.** Figures A) and B) show a histological section of a SAMR1 mouse ileum and an XPCT tomogram, respectively. The histological section was stained with haematoxylin and eosin to highlight nuclei, extracellular matrix and cytoplasm, and alcian blue for goblet cells. By comparing the images, it is possible to identify the morphological structures in the XPCT tomograms with certainty: goblet cells (indicated by white arrows), epithelial cells (EC), lamina propria (LP), longitudinal (1) and circular (2) layers of the tunica muscolaris. XPCT experiment performed at the ANATOMIX beamline (Soleil). In the greyscale of these XPCT images, light shades correspond to highly absorbing features and dark shades to low absorbing tissues.

Table S1. Villus length data. Data used for the quantification of villus lengths shown in Figure 1.

Table S2. Crypt depth data. Data used for the quantification of crypt depth shown in Figure 1.

Table S3. Goblet and Paneth cells data. Data used for the quantification of the number of goblet and Paneth cells per crypt shown in Figure 4.

Table S4. Isolated Lymphoid Follicle density. Data used for the quantification of the density of isolated lymphoid follicles shown in Figure 6.

Movie S1. 3D rendering of a murine ileal crypt. The rendering was obtained from nano-X-ray phase contrast tomography (XPCT) data acquired at the ESRF beamline ID16A. The high quality of the tomographic images makes it possible to clearly distinguish the morphological features of the crypt structure, allowing the segmentation of the epithelial layer of a crypt (rendered in dark green), together with the goblet cells (light blue), whose apical portion expands with mucin-secreting granules and extends into the intestinal lumen, and the Paneth cells, characterized by their typical pyramidal shape with prominent apical granules (yellow) occupying most of their cytoplasmic region.
